# Wall-type and indoor residual spraying application quality affect the residual efficacy of indoor residual spray against wild malaria vector in southwest Ethiopia

**DOI:** 10.1186/s12936-018-2458-3

**Published:** 2018-08-20

**Authors:** Zerihun Desalegn, Teklu Wegayehu, Fekadu Massebo

**Affiliations:** Department of Biology, Arba Minch University, Arba Minch, Ethiopia

**Keywords:** Propoxur, Routine spray, Standard spray, Wall surface type

## Abstract

**Background:**

Residual efficacy of indoor residual spray may vary with different spray quality and wall surfaces types. This study evaluated the impact of spray quality and wall surface types on residual efficacy of propoxur against wild *Anopheles gambiae* sensu lato (s.l.) in southwest Ethiopia.

**Methods:**

Thirty houses of different mud wall surfaces (10 smooth, 10 rough, 10 painted) were selected and randomly allocated into routine and standard spray. The routine spray was conducted by district health office as usual, while the standard spray was done by strictly following guidelines. Three control houses were selected from unsprayed nearby semi-urban. Wild *An. gambiae* s.l. were used for wall bioassay tests. Two-way mixed model analysis of variance was used to analyse the data. The mean variation between wall and spray types was compared by post hoc analysis of IBM SPSS version 20.

**Results:**

On standard spray, knockdown rate was 95.3% on painted, 82% on smooth and 72.5% on rough surface at week 17 of post-spray, whereas on routine spray it was 82.7% on painted, 48.7% on smooth and 60% on rough surface. On standard spray, mortality rate of *An. gambiae* s.l. was 99.3% on painted surface, 90% on smooth and 80% on rough surface. On routine spray, it was 89.3% on painted, 61.3% on smooth and 65% on rough surface at week 17 of post-spray. The painted wall surface showed the highest knockdown rate (86.4–100%) on standard and (73.8–91.5%) routine spray; mortality rate was more than 80% on both spray types during the 17 weeks of follow-up regardless of spray types. The lower mortality rate and residual effect was observed on routine smooth and rough wall surfaces. The residual efficacy of propoxur was > 80% at week 17 on standard spray regardless of the wall types and it was < 80% on routine spray except painted wall surface.

**Conclusion:**

The painted wall surface and standard spray showed better residual efficacy. Therefore, it is recommended to consider the wall surface available in the community to estimate the residual lifespan of the insecticide, and strictly to follow the spray guideline to improve the effectiveness of indoor residual spray.

## Background

Malaria is spread from one person to another by female mosquitoes of the genus *Anopheles* and is one of the major public health problems of the world with an annual estimate of 212 million cases and 429,000 deaths, mainly in the sub-Saharan Africa, in 2015 [1]. It was accountable for about 303,000 malaria deaths in under-five children globally in 2015, and 10% of under-five deaths in sub-Saharan Africa. On the other hand, malaria case incidence decreased by 41% and mortality by 62% globally between 2000 and 2015 [1].

Long-lasting, insecticide-treated nets (LLINs) and indoor residual spray (IRS) are the two cornerstone malaria control interventions contributing to current malaria reduction [2]. The two intervention tools are effective against indoor resting and biting malaria vectors [3]. In the 1950s, dichlorodiphenyltrichloroethane (DDT)-based IRS was initiated and implemented to control malaria in Ethiopia [4]. It was the principal tool in the 1950s’ and 1960s’ malaria eradication programme. Its use was discontinued in 2009 and was replaced by pyrethroid insecticides, due to the widespread distribution of DDT-resistant malaria vectors [5]. In 2011, pyrethroid-based IRS was replaced by carbamate, due to the high level of resistance in malaria vector populations. Currently, carbamate insecticides are in use for IRS and the principal malaria vector, *Anopheles arabiensis* is susceptible to these insecticides in most parts of the country [5, 6].

The insecticide sprayed on the wall surface should be stable in order to minimize the number of spraying cycle in the targeted malaria transmission seasons. In areas where the transmission season is more than 6 months, multiple spraying can be required and become expensive due to the high demands of logistics [7]. However, residual efficacy of IRS may be varied with wall type and the quality of spray application [8, 9]. Moreover, the effectiveness of IRS depends on resistance status of malaria vectors and its residual time on the wall surface [9]. Poor quality application may contribute to insecticide resistance and could be a challenge for malaria vector control [10]. If the residual effect of IRS is shorter than expected, it may contribute to an increase in incidence of malaria infection where the malaria transmission season exceeds the residual effect of insecticides [11].

There are different wall surfaces in the study area and it would be useful to determine the residual life of insecticide on different wall surface. There is little information on the effect of wall types and application quality on the residual efficacy of carbamate insecticides used for IRS in Ethiopia [12]. Therefore, the impact of wall type and application quality on the efficacy of propoxur was assessed against the principal malaria vector, which might help to modify the IRS implementation programme.

## Methods

### Description of the study area

Gamo Gofa is one of 13 zones in the Southern Nations Nationalities and Peoples Region (SNNPR). Arba Minch, the capital of Gamo Gofa, is located 505 km southwest of Addis Ababa and 275 km from Hawassa, the regional capital city. Malaria is one of the public health problems in the Arba Minch Zuria district. Shellie Mella is among the 11 malarious villages in the district. This village was selected purposely, based on its malaria endemicity and accessibility for study. It is located 20 km south of Arba Minch at an altitude of 1120–1380 m above sea level (masl). The annual rainfall is 900–1300 mm and its annual temperature is 25–36 °C. The total population of the village was 10,721 (5253 males and 5468 females) within 2188 households. The main source of income is agriculture (banana and mango cultivation).

The population lives in houses with different wall surfaces, including rough, painted or smooth mud walls with grass thatched or iron roofs. There is one governmental health centre and one health post in the study area. IRS and LLINs are the two major vector control interventions implemented by the District Health Office. The coverage of the IRS was 98% and that of LLINs was 99% in 2016.

### Study design

This experimental trial was carried out in Shellie Mella from August to November 2016. The list of houses was taken from health post and 30 houses were selected randomly from the community with different wall types (10 smooth, 10 painted, 10 rough mud walls). The houses were then coded and assigned randomly into two arms before spraying. Fifteen houses (five from each wall type) were assigned for standard spray and the other 15 houses (five from each wall type) for routine spray. The housing condition of the two groups was characterized to make sure that the houses are comparable in most characteristics other than application type to minimize bias. Three control houses with the three wall types (rough, painted, smooth mud wall) and comparable characteristics to the intervention houses were selected from unsprayed nearby semi-urban village. Control houses were used for adjustment of percentage mortality of mosquito by Abbott’s formula [13]. Spray operators, supervisors and data collectors were blinded to spray type allocation and bioassay test to minimize biases. Verbal and written consent was obtained from head of households before spraying. The routine spray was coordinated by District Health Office and conducted as usual by spray operators. Standard spray was conducted by strictly following the WHO and national spray guideline using the same spray operators.

### Test mosquito rearing procedure

The larvae and pupae of *Anopheles* mosquitoes were collected from Kulfo River and surrounding areas and then transported to Arba Minch University Medical Entomology laboratory. Pupae were separated using pipette and placed into adult mosquito cages. Adults were provided with cotton wool soaked in 10% sugar solution. The insectary environment was maintained at temperature of 27 ± 2 °C and relative humidity of 60 ± 10%. Then, 3–5 days old sugar-fed female *Anopheles gambiae* s.l. were used for wall bioassay tests.

### Insecticide susceptibility tests

Insecticide susceptibility test was carried out in the laboratory against the wild female *An. gambiae* s.l. using WHO test kit and 0.1% propoxur-impregnated paper. This test was done to ensure susceptibility of the species before conducting cone bioassay tests. The procedure was carried out according to WHO guidelines [13]. Four replicates of 25 female mosquitoes were exposed to insecticide-impregnated test paper in each tube for 1 h. Two replicates of the same batch of mosquito were exposed on oil-impregnated papers for control. Mortality was recorded after 24 h of exposure. The resistance status of *An. gambiae* s.l. was determined according to the latest WHO [13] criteria as follows:

- Mortality rates between 98 and 100% indicate full susceptibility.
- Mortality rates between 90 and 97% require further investigation.
- Mortality rates < 90%, the population considered resistant to the tested insecticide [13].

*Anopheles gambiae* s.l. were fully susceptible to propoxur 0.1% with 100% mortality after 24 h.

### Inclusion and exclusion criteria

All houses with smooth, rough and painted mud walls were eligible for the study. Householders who declined informed consent, newly constructed houses and where mud walls were not dry during the selection were excluded. Houses with humans and animals living together were excluded as it is unusual to share houses with animals in the area. Moreover, houses were excluded if kitchen and living room were one to minimize the impact of smoking on insecticide.

### Indoor residual spraying application

#### Routine and standard spraying application

Those houses selected and coded for routine spray were sprayed by trained community spray operators during the routine spray schedule of the area. The spray was coordinated by, and training given for 6 days to spray operators by District Health Office. Propoxur 50% water-dispersible powder (WP) available under trade name of FICAM^®^ was supplied by District Health Office.

The other 15 houses (5 smooth, 5 painted, 5 rough mud walls) randomly selected for the standard spray were sprayed with the same chemical deployed in the community. Two local spray operators from those involved in routine spray were used for standard spray. The difference was that in standard spray the operators strictly followed the WHO and national spraying operation guidelines [9, 14]. Spraying was conducted in collaboration with District Health Office. Additional orientation on standard spraying operation was given by IRS experts from the District Health Office. Spray equipment, personnel protective clothing, goggles, gloves and other materials for safety of spray operators were obtained from District Health Office. The insecticide application was done at rate of 2 g/sq m in the form of a WP [15] using a Hudson X-pert^®^ sprayer (8-l capacity) with HSS-8002E nozzle, which was equipped with a regulator adjusted at angle of 80° and discharging rate of 760 m per minute at a standard tank pressure of 55 psi.

The spray operators strictly followed the instructions on the product label to ensure safe and correct mixing (one sachet or 500 g in 8 l water), handling and application of insecticides. The insecticide was mixed outdoors in well ventilated areas [9]. Before starting spraying, information was provided for householders about the safety, purpose and time of spraying. The inhabitants were requested to prepare and remove all materials such as water, food and cooking utensils from the houses before spraying. Household members were allowed to enter the house 2 h after spraying. The inhabitants were also requested not to wash, paint or re-plaster the sprayed walls [9]. Spray operators also reassured householders about the safety of insecticide being used, and that applied insecticides did not damage walls, ceilings and furniture.

#### Cone bioassay test using wild *Anopheles gambiae* s.l.

The wall bioassay tests were carried out by using the standard WHO cone method. The first bioassay was carried out after 1 day of spraying, then after 1 week and then every 4 weeks for 4 months from August to November 2016. Each day, both spray types were conducted on 15 houses of three wall surfaces. Three WHO cones with 12-cm diameter were firmly fixed on each wall surface for both standard and routine sprayed houses at three different locations (lower, middle and upper) above the ground [9].

Then, 10 3–5 days old female *An. gambiae* s.l. were introduced into each cone fixed on the wall by using mouth aspirator. At the same day, separate cones were fixed on control houses and the same number of mosquitoes was introduced using separate mouth aspirator to avoid contamination. After 30 min of exposure, the mosquitoes were transferred into plastic cups and 10% sugar was provided. The temperature was maintained at 27 ± 2 °C and relative humidity was 70 ± 10% for 24 h. The percentage of knockdown after 1 h of exposure was recorded. Mortality rate was recorded after a 24-h holding period.

### Safety procedures

On the day of spraying all family members were advised to remain out of rooms for 2 h after spraying to avoid any possible risk during and after the spraying of their houses. The adults of household were also advised to tell their children not to touch the sprayed walls for at least 1 day after spraying. For environmental safety, the containers or sachets were returned to District Health Office for proper disposal [9]. Both spray operators and household members were informed of signs of adverse effect and advised to report any adverse effect of insecticide during 1 week post-spraying and no adverse effect was reported from either household members or spray operators during spray time and 1 week after spraying.

### Outcome variables

The first primary outcome variable was the time to knockdown of *An. gambiae* s.l. within 30 min of exposure on different wall surfaces and spray types. The second primary outcome variable was the percentage the mortality rate after 24 h holding period during the follow-up period.

### Data analysis

Knockdown and mortality rates were calculated and analysed according to WHO protocol to determine the efficacy of IRS. Percentage of knockdown was calculated after 30 min exposure. The percentage mortality was calculated after 24 h holding period. All mosquitoes that could fly were considered alive. Control mortality was less than 5% in all bioassay during the study period and hence was not corrected by Abbott’s formula.

Two-way mixed model ANOVA was used to determine the mean knockdown and mortality variation among the wall surfaces and spray types. Post-hoc test analysis was employed to identify spray type and wall type with significant difference. A significance test was done by *p* value < 0.05. Treatment was considered effective until mortality rate in exposed mosquitoes was ≥ 80% within 24 h [16]. Two houses (one standard and one routine rough wall surfaces) were excluded from analysis as they were replastered. Data were entered and analysed using IBM SPSS version 20 (SPSS Inc, Chicago, USA).

## Results

### Knockdown rate on different spray and wall types

The knockdown rates of *An. gambiae* s.l. after 1 h exposure varied on standard and routine spray of different wall surfaces (Tables 1 and 2). The knockdown rate was 100% for all wall surfaces in both spray types on the first week of bioassay. On both spray types, painted wall surface revealed 100% knockdown rate for 13 weeks and more than 80% at week 17, while it was low on smooth and rough wall surfaces of both spray types. At week 17 of the standard spray, the knockdown rate was 95.3% (95% confidence interval (CI) 86.4–100) on painted, 82% (95% CI 73–90.9) on smooth, and 72.5% (95% CI 62.6–82) on rough surface. Whereas on routine spray, the knockdown rate was 82.7% (95% CI 73.8–91.5) on painted, 48.7% (95% CI 39.8–57.5) on smooth, and 60% (95% CI 50–69.9%) on rough surface.

**Table 1:** Mean mosquito knockdown rate on different wall surface by standard spray through time post spray in Shellie Mella, southwest Ethiopia

Wall types	Time in weeks					
	Day 1	Week 1	Week 5	Week 9	Week 13*	Week 17*
Painted	100	100	100	100	100	95.3
Smooth	100	100	100	98.7	99.3	82
Rough	100	100	96.7	87.5	78.3	72.5

* Significant difference (P < 0.05)

**Table 2:** Mean mosquito knockdown rate on different wall types by routine spray through time post spray in Shellie Mella, southwest Ethiopia

Wall types	Time in weeks					
	Day 1	Week 1	Week 5*	Week 9*	Week 13*	Week 17*
Painted	100	100	100	100	100	82.7
Smooth	100	100	84	71.3	82	48.7
Rough	100	100	87.5	78.3	68.3	60

* Significant difference (P < 0.05)

The mean knockdown rate of mosquito was significantly different among spray types applied (F(1,78) = 44.6, P < 0.001). Also it was different among different wall surfaces sprayed (F(2,78) = 32.5, P < 0.001). The significant difference occurred between standard painted and standard rough wall surface (P < 0.001) (Table 3) and standard smooth and rough starting week 13 (P < 0.0001) (Table 1).

**Table 3:** Mean mosquito knockdown rate on wall types and spray type at 17 weeks bioassay in Shellie Mella, southwest Ethiopia

Wall type	Spray type	N	Mean % knockdown	95% CI	SE	P-value
Painted	Standard	15	95.3^a^	86–100	4.5	0.37
	Routine	15	82.7^a^	72.9–91	4.5	
Smooth	Standard	15	82^a^	73.1–90.9	4.5	< 0.001
	Routine	15	48.7^b^	39.8–57.7	4.5	
Rough	Standard	12	67.5^c^	58–76.4	4.9	0.9
	Routine	12	64.4^b,c^	52.7–76	4.9	

Change in letters between columns indicates statistically significant difference between wall and spray types

There was no significant difference between standard painted and standard smooth throughout the follow-up period (P = 0.9). However, on routine spray, the significant difference occurred between painted and smooth, and painted and rough starting week 5 (P < 0.0001) (Table 2). But, there was no difference between rough and smooth throughout the follow-up period (P > 0.05) on routine spray. The post hoc test analysis showed that the highest knockdown rate was observed on painted wall surface irrespective of type of spraying (83–100%). However, the knockdown rate was not varied in painted wall surface ranging between 95 and 100% on standard and 83–100% on routine spray (P = 0.37). In the control houses there was no mosquito knockdown observed after 1 h of exposure in all wall surfaces.

### Mortality rate on different spray and wall types

The mortality rate of *An. gambiae* s.l. after 24 h holding time on different wall types by standard and routine spray application is indicated in Tables 4, 5. More than 80% mortality rate was observed on standard spray on all wall types during the 17 weeks bioassay tests. On routine spray, it gradually decreased from 100 to 61% on smooth and 100 to 65% on rough wall types. The mortality rate of *An. gambiae* s.l. was above 80% on painted wall even in routine spray at 17 weeks.

**Table 4:** Mean mortality rates of mosquito on different wall surface by routine spray in Shellie Mella, southwest Ethiopia

Wall types	Time in weeks					
	Day 1	Week 1	Week 5	Week 9*	Week 13*	Week 17*
Painted	100	100	100	100	100	89.3
Smooth	100	100	98.7	82.3	82	61.3
Rough	100	100	100	91.7	87.5	65

* Indicates significant difference (P < 0.05)

**Table 5:** Mean mortality rates of mosquito on different wall surface by standard spray in Shellie Mella, southwest Ethiopia

Wall types	Time in weeks					
	Day 1	Week 1	Week 5	Week 9	Week 13	Week 17*
Painted	100	100	100	100	100	99.3
Smooth	100	100	100	100	100	90
Rough	100	100	100	100	97.5	80

* Indicates significant difference (P < 0.05)

There was a significant difference in mean mortality rate of *An. gambiae* s.l. between spray types (F(1,78) = 58.27, P < 0.001). Standard spray showed better mosquito mortality rate than routine spray during 17 weeks of assessment on all three wall types. There was also significant difference in the mean mortality rate between the wall types (F(2,78) = 26, P < 0.001). Post-hoc test showed that the highest mortality rate was on painted mud wall surface. On standard spray, the difference on mortality rate was observed only between painted and rough surface at week 17 (P < 0.001), but no difference was seen between painted and smooth wall types (Table 6). On routine spray, the significant difference was occurred from week 9 (Tables 4, 5). However, mortality rate was not significantly varied between smooth and rough mud wall types on both spray types (P = 0.83). Also, mortality rate was not significantly varied on painted wall surface on standard (92.8–100%) and routine (82.8–95.8%) spray types (P = 0.84). In the control houses, mosquito mortality rate after 24 h recovery period was less than 5% in all wall types.

**Table 6:** Mean mortality rate of mosquito on different wall surface versus spray type at week 17 in Shellie Mella, southwest Ethiopia

Wall type	Spray type	N	Mean % mortality	95% CI	SE	P-value
Painted	Standard	15	99.3^a^	92.8–100	0.93	0.84
	Routine	15	89.3^a^	82.8–95.8	0.93	
Smooth	Standard	15	90^a,c^	83.5–96.5	0.93	< 0.001
	Routine	15	61.3^b^	54.8–67.8	0.93	
Rough	Standard	12	80^c^	72.7–87.3	1.04	< 0.001
	Routine	12	65^b^	57.7–72.3	1.04	

Change in letters between columns indicates statistically significant difference between wall and spray types

### Residual time of propoxur on different spray surfaces and wall types

**Fig. 1:**
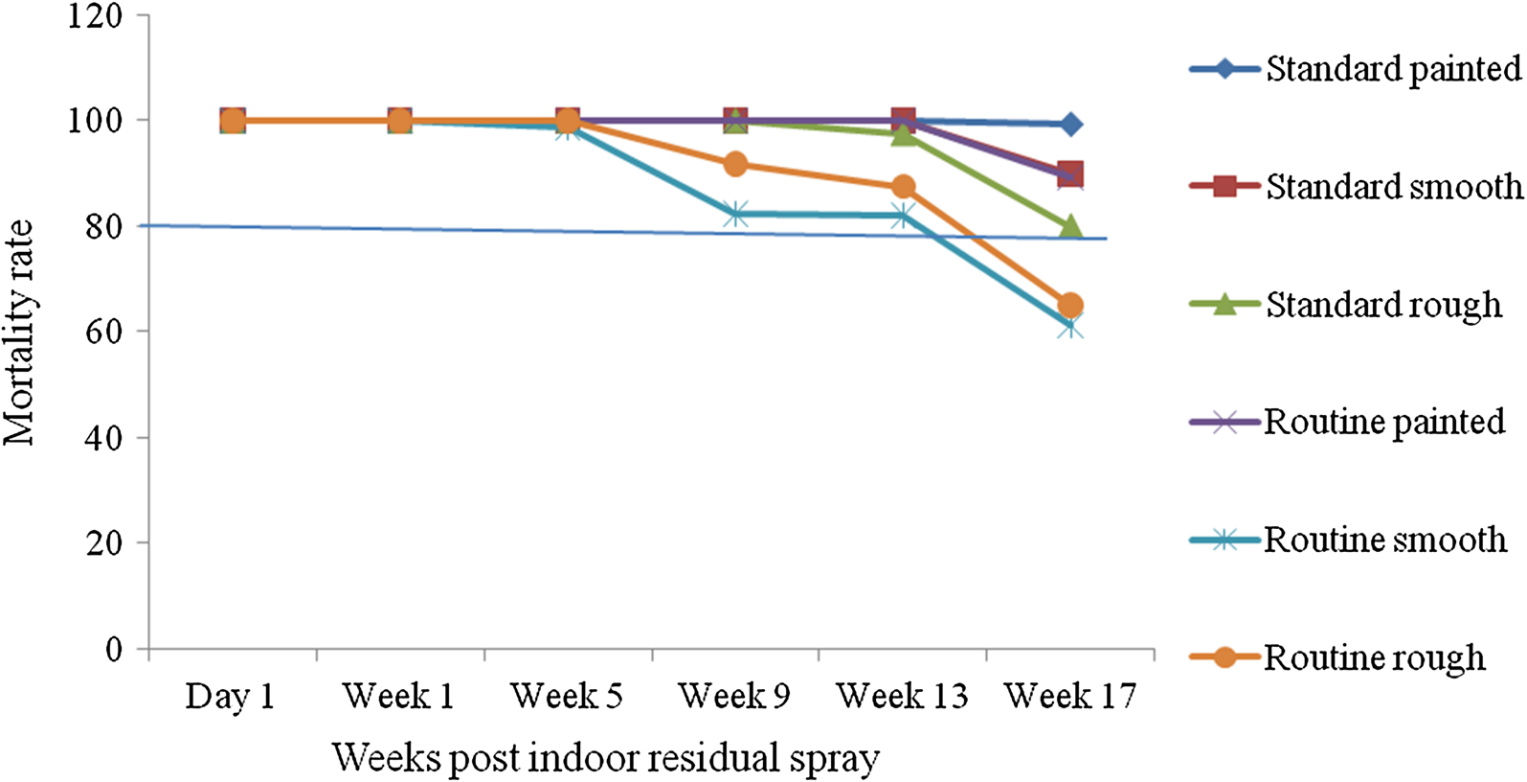
WHO cut-off point (mortality rate ≥ 80%) on different spray and wall types during the 17 weeks follow-up period in Shellie Mella, southwest Ethiopia

The residual effect of propoxur was defined by mortality rate based on the WHO cut-off point which was > 80%. The mortality rate of *An. gambiae* s.l. was > 80% in both spray and wall types until week 13. However, the mortality rate on routine smooth and rough surface was < 80% at week 17 (Fig. 1). The residual time of propoxur was more than 17 weeks for standard spray irrespective of the wall types. In routine spray, the residual time was more than 17 weeks on painted wall surface only.

## Discussion

This study indicated that the spray quality and wall types in the community affect the residual efficacy of IRS. The knockdown rate of *Anopheles* mosquito was varied in different spray type and wall surface. The knockdown rate was higher on standard painted wall, while it was lowest in routine smooth. The mortality rate of *An. gambiae* s.l. was high on standard painted wall and lowest on routine smooth. The residual duration of propoxur was influenced by spray and wall types. On standard spray, it was more than 17 weeks for all three wall types, while it was less than 17 weeks for routine spray except painted wall surface.

The standard spray and painted wall surface showed better knockdown rate. The experimental study conducted in Cameroon assessed the knockdown rate of *An. gambiae* sensu stricto (s.s.) exposed to bendiocarb-treated wood, concrete and mud wall surface [10]. They reported that more than 98% knockdown rate during 13 weeks observed on wood and concrete wall surface, while it was 80% on mud wall surface and there was statistically significant difference at p < 0.05. Although the wall types used in that study were different from the present study, it showed that knockdown rate was affected by wall types. A study conducted in Karagwe district in Tanzania on residual effect of lambdacyhalothrin (capsule suspension) insecticide reported 100% knockdown rate on painted surface in 3 months follow-up period, however, it was only 35% on mud surfaces [17]. Similarly in Muleba district, the highest knockdown rate was observed on painted surface (67%) and the lowest on mud substrate (45%) during 3 months follow-up [17]. This was consistent with the present study finding which showed the impact of wall type on knockdown rate and better knockdown rate observed on painted surfaces.

In current study, the standard spray showed better mortality rate on all wall surfaces. Also, painted wall surface has better effect on mortality of mosquito in both spray types. Better performance (100% mortality rate) of bendiocarb sprayed on painted walls for up to 6 months was documented in Ethiopia, while the residual performance was significantly lower in dung and mud-plastered wall surfaces [18]. Another study in Adulala village in Ethiopia showed the highest mortality of mosquitoes on bendiocarb-sprayed, painted wall surface than others, but the difference was not significant [19]. The possible explanation for high performance of the painted wall surface for long periods could be due to the closing of small pores that pass insecticide through and may reduce the biodegradability of insecticide on walls. On the other hand, smooth and rough mud walls also have pores that may pass insecticide and may reduce efficacy of the insecticide.

Based on WHO recommendations [13], an ideal insecticide should have a minimum residual effect of greater or equal to 80% mosquito mortality after 24 h post-exposure during the recommended lifetime. In this finding the residual time of propoxur on standard spray was more than 17 weeks in all three wall types, which is in line with WHO recommended estimated time [15]. However, the residual effect of propoxur did not assess for 6 months on standard spray of all wall types and painted wall surface of routine spray as the mortality was > 80%. The residual efficacy of propoxur on smooth and rough wall surface was less than 17 weeks, which was below the WHO recommendation [15]. Therefore, based on this finding, spray cycle may not exceed 17 weeks for routine spray on smooth and rough wall surfaces. The performance of the residual efficacy of propoxur was higher on painted wall surfaces than dung and non-painted mud wall surfaces [9].

Another study by Oxborough and his colleagues reported shorter residual efficacy of deltamethrin on mud wall than concrete against *An. arabiensis* both in the laboratory and field tests [20]. The study done by Yewhalaw et al. [21] documented higher performance of propoxur on the painted wall type against the laboratory colony of *An. arabiensis*. On the contrary, maximum persistence of bendiocarb against *Anopheles culicifacies* was observed on unpainted mud walls, followed by brick and cement walls [22]. However, the residual life of propoxur was little affected by wall types [18]. This difference may be due to difference in chemical nature of the wall surfaces or strains of mosquito species, the pH of the wall and local temperature or relative humidity.

This study has several strengths. There are limited studies on impact of wall and spray types on residual effect of insecticide at community level in Ethiopia, and the current study may provide evidence to improve vector control. Wild populations of malaria mosquitoes were used for bioassay, which may reflect the actual status in the community as recommended by WHO guidelines. The limitations include the failure to follow the complete WHO recommended duration (up to 6 months) and to conduct quality control test by high performance liquid chromatography due to absence of reagent and dye-functionality of machine. The effect of local temperature, humidity and pH of the wall surface were not considered in this study as these factors may affect the residual efficacy of propoxur.

## Conclusions

Wall surface types and spray application quality has an impact on the residual efficacy of propoxur IRS. Propoxur has the highest knockdown and mortality rate of standard spray on all wall surfaces and painted wall surfaces of both spray types. It showed better residual efficacy when applied by strictly following WHO guideline and on painted wall surface types. Therefore, wall surface type available in the community could be considered during decision-making of an IRS programme. IRS could be conducted using national and WHO guidelines to improve residual efficacy of insecticides. Awareness of the importance of painting houses on the residual effect of insecticides could be included in health education programmes. Moreover, research is recommended on impact of spray quality and wall types on residual effect of different insecticides in wide geographic areas.

## Abbreviations

a.i: active ingredient
CS: capsule suspension
EC: emulsifiable concentrate
WG: water-dispersible granule
SC: suspension concentrate
